# Membrane Recruitment of Scaffold Proteins Drives Specific Signaling

**DOI:** 10.1371/journal.pone.0000977

**Published:** 2007-10-03

**Authors:** Frédéric Pincet

**Affiliations:** Laboratoire de Physique Statistique, Ecole Normale Supérieure, Paris, France; Ordway Research Institute, United States of America

## Abstract

Cells must give the right response to each stimulus they receive. Scaffolding, a signaling process mediated by scaffold proteins, participates in the decoding of the cues by specifically directing signal transduction. The aim of this paper is to describe the molecular mechanisms of scaffolding, i.e. the principles by which scaffold proteins drive a specific response of the cell. Since similar scaffold proteins are found in many species, they evolved according to the purpose of each organism. This means they require adaptability. In the usual description of the mechanisms of scaffolding, scaffold proteins are considered as reactors where molecules involved in a cascade of reactions are simultaneously bound with the right orientation to meet and interact. This description is not realistic: (i) it is not verified by experiments and (ii) timing and orientation constraints make it complex which seems to contradict the required adaptability. A scaffold protein, Ste5, is used in the MAPK pathway of *Saccharomyces Cerevisiae* for the cell to provide a specific response to stimuli. The massive amount of data available for this pathway makes it ideal to investigate the actual mechanisms of scaffolding. Here, a complete treatment of the chemical reactions allows the computation of the distributions of all the proteins involved in the MAPK pathway when the cell receives various cues. These distributions are compared to several experimental results. It turns out that the molecular mechanisms of scaffolding are much simpler and more adaptable than previously thought in the reactor model. Scaffold proteins bind only one molecule at a time. Then, their membrane recruitment automatically drives specific, amplified and localized signal transductions. The mechanisms presented here, which explain how the membrane recruitment of a protein can produce a drastic change in the activity of cells, are generic and may be commonly used in many biological processes.

## Introduction

### The network of signaling pathways, from complexity to universality

A network of pathways is used by cells in order to detect, analyze and respond to external stimuli. This network is characterized by two somewhat contradictory features: complexity and universality.

Complexity comes primarily from the imbalance between the great diversity of potential stimuli and the limited number of signaling pathways available to the cell [1], [2]: there cannot be any one-to-one relation between stimuli and signaling pathways. Many tricks are used by cells to code signaling and make it unambiguous, *e.g.* simultaneous activation of several pathways or molecules, the time and/or location where the pathway is activated,… During evolution, each species managed to create its own unique complex signaling code.

It is surprising that, in spite of this uniqueness and complexity of each code, universal signaling schemes can be found in various species. For instance, some generic pathways that have been recognized as major actors for the specificity of the mediated signal are conserved in organisms and cell types that range from yeast to mammals [3]. This means that each organism was able to adapt these pathways for its own purpose. To understand how this adaptability was achieved, it is necessary to describe the generic means of action of the molecules involved in these pathways. These generic features are the basis of signaling. The purpose of this paper is to present one of these universal features, scaffolding, which is ensured by scaffold proteins that bind several proteins involved in the same signaling pathway. It has been long recognized that scaffolding plays an important part in the specificity of the cell response to some stimuli [4]–[6]. Even though it is commonly assumed that several molecules of a pathway can simultaneously be bound to scaffold proteins with the correct orientation to meet and interact, the molecular mechanisms of scaffolding are still unclear and have been questioned for years [7]. Here, it will be shown that this common picture is not appropriate and the generic principles of the actual mechanisms of scaffolding will be presented. These mechanisms are extremely simple and efficient: the sole membrane recruitment of scaffold proteins is sufficient to drive a specific, amplified and localized response of the cell.

## Analysis

### Phosphorylation/dephosphorylation cycle

Signaling pathways consist in the serial activation of several molecules, mainly proteins, by chemical reactions that change their conformation. A typical reaction inducing conformational changes is the phosphorylation of a protein by a kinase [8], [9]. Here, we will focus on signaling pathways in which kinases are activated by phosphorylation in series.

The phosphorylation/dephosphorylation cycle through which a protein oscillates between a basal state, S, and a fully phosphorylated state, S*, may comprise several intermediate states. These intermediate states make the cycle extremely difficult to analyze and quantify because they add a large number of parameters to the molecular system. In the method section, we show that it is possible to simplify the cycle provided that two reasonable assumptions are made: low concentrations of the intermediate states and steady state regime. Then the cycle can be written as a single chemical reaction:
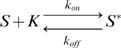
(1)Besides, the conservation of matter leads to (see method section):
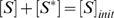
(2)Where [S]_init_ is the initial value of [S].

Reaction (1) and equation (2) along with the steady-state hypothesis show that each cytosolic substrate activated by a cytosolic kinase follows a reaction-diffusion equation in the cytosol:
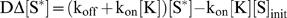
(3)Δ is the Laplace operator.

When the kinase is present only at the membrane, the reaction-diffusion equation reduces to:
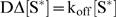
(4)For a given pathway the reaction-diffusion equations corresponding to each kinase/substrate pair have to be considered simultaneously. The overall system will be fully defined once the boundary conditions at the membrane, which represent the source of active proteins at the membrane, are known. These boundary conditions can easily be obtained in the case of scaffold proteins and have a strong influence on the cell response (see method section).

### The postulated parts of scaffold proteins in cell signaling

Since there is a great variety of stimuli, pathways must often be used to convey several signals. Scaffold proteins are commonly employed by cells in order to direct the pathway towards the correct target.

In this section, we will present the postulated molecular mechanisms of scaffolding.

### The reactor model vs. adaptability

**Figure 1:**
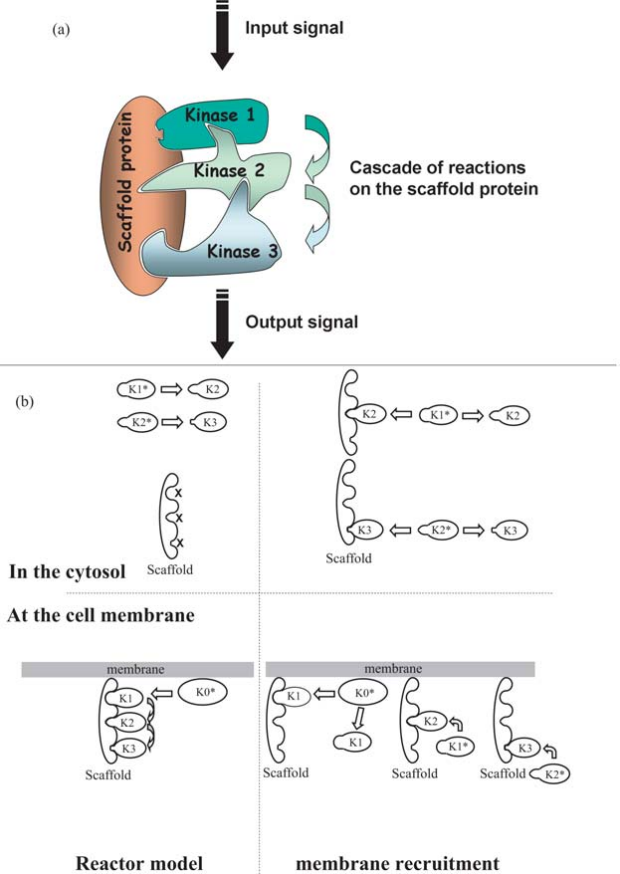
How a scaffold protein works. 1a: Reactor model In the reactor model, the scaffold protein is able to simultaneously bind several kinases (three in this figure) that will activate each other in series very quickly. The scaffold protein is a reactor where the kinases are correctly oriented in order to facilitate the reactions. 1b: Constraints in the reactor model and in the simple membrane recruitment. A cascade of 4 kinases is represented: K0, K1, K2 and K3. K0 is assumed to be membrane bound (like Ste20 in the case of the MAPK pathway studied in the text). The arrows represent the activation of a kinase by another one (* indicates that the kinase is active). In the reactor model, the successive kinases, K1, K2 and K3, bind, meet and interact onto a scaffold protein (noted “Scaffold”). This model requires many geometrical and energetic constraints. It also implies that the binding sites for the various kinases on the scaffold protein must be blocked in the cytosol to prevent the scaffolding process to happen in volume. In the simple membrane recruitment, the scaffold protein binds independently the three kinases: when in the cytosol, it does not affect the signal transduction; when recruited to the membrane, it creates a source of active molecules at the membrane. The only constraint in this description is that the binding site between two successive kinases has to remain accessible when the kinase to be activated is bound to the scaffold protein.

As suggested by their name, scaffold proteins are classically considered like reactors where several molecules of a pathway can simultaneously be bound with the correct orientation to meet and interact in order to direct the pathway towards a specific response of the cell [10], [11]. In this reactor picture (see Fig. 1), the cascade of reactions of the pathway happens directly on the scaffold protein itself.

Similar scaffold proteins are found in many species. Hence, it evolved differently according to the own purpose of each organism. This means scaffold proteins require adaptability. The reactor model imposes many constraints which are in contradiction with the requirement of a system that is highly adaptable throughout evolution: these constraints make scaffold proteins not easily adaptable and therefore a different scaffold protein has to be redesigned for each application and species.

First, there are geometrical constraints since the molecules bound to the scaffold protein need to have the right orientation in order to interact. There are also energetic constraints because the molecules have to be bound to the scaffold protein long enough to meet their interacting partners but not too long in order to propagate in the cell when activated and free the scaffold protein binding site. Finally, there is a chemical constraint: the scaffold protein must be inactive in the cytosol, otherwise, their membrane recruitment is useless for the specificity of the signal. Thus, the binding sites for the molecules of the pathway must be blocked when the scaffold protein is in the cytosol.

Besides, we will show in the discussion that, at a given time, there is usually no more than one molecule bound to the scaffold protein. Therefore the reactions cannot occur in series on the scaffold protein.

Finally, we will show that the reactor model is not confirmed by experiments.

Hence, this reactor model is not realistic and the actual molecular mechanisms of scaffolding still remain to be described.

### Ste5 in *Saccharomyces cerevisiae*

To analyze the mechanisms of scaffolding, we will use a widely spread example [12] of pathway used in several signaling processes: the mitogen-activated protein kinase (MAPK) pathway [13]–[15].

**Figure 2:**
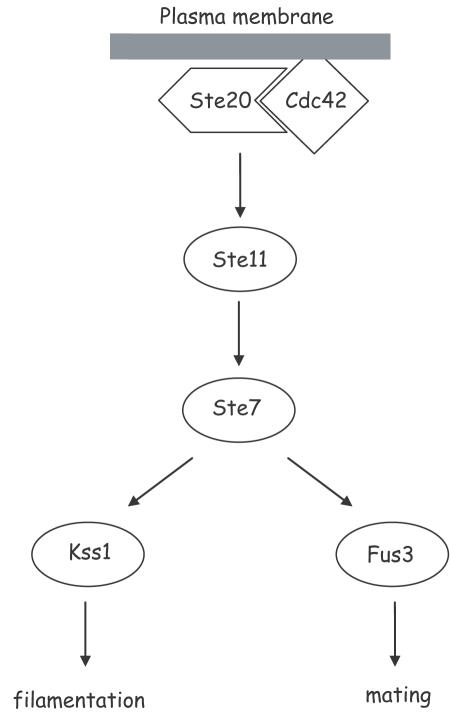
MAPK pathway in *Saccharomyces Cerevisiae.* The MAPK pathway in *Saccharomyces cerevisiae* consists in a cascade of four successive kinases: Ste20, Ste11, Ste7 and a MAPK. In the results presented in the text, two MAPK are studied, Fus3 and Kss1. Activation of Kss1 has been recognized to be predominant during filamentation while Fus3 is relevant for the mating response. During mating, a scaffold protein, Ste5 is recruited to the membrane and bind to Ste11, Ste7 and Fus3. When activated, the Rho family protein cdc42, a small GTPase localized to the plasma membrane, binds and activates Ste20 through a CRIB domain. Ste20 remains at the plasma membrane. This point is the start of the cascade of reactions where a kinase activates the next kinase by phosphorylation. Ste20 activates the MAPK kinase kinase Ste11, which in turn activates a MAPK kinase, Ste7, which activates two different MAPKs, Kss1 and Fus3.

In the yeast, *Saccharomyces cerevisiae*, this pathway starts with the phosphorylation of the MAPK kinase kinase Ste11 by the p21-activated kinase (PAK) Ste20. Active Ste11 phosphorylates the MAPK kinase Ste7 that will in turn phosphorylate two MAPKs, Fus3 and Kss1 (see Fig. 2) [1], [16]–[18]. The MAPK pathway has already been extensively studied and a massive amount of quantitative data has been obtained [2], [19]–[23]. The association and dissociation constants and the overall numbers of each type of protein have been previously estimated [24]–[27]. This allows us to write and solve the complete set of reaction-diffusion equations for Ste11, Ste7, Fus3 and Kss1 in the cytosol.

The roles of the two MAPKs, Kss1 and Fus3, are fundamentally different in the cell life. It has been shown that Fus3 plays its main part in the response to mating pheromone and Kss1 during filamentation induced by nutrient limitation [1], [2], [15], [18], [28]. Even though the same pathway is activated for both stimuli, it is critical for the cell to give the right response: it is not time for mating when there is no food.

This is where scaffold proteins intervene. Even though the same pathway is used for mating and filamentation, during mating, a heterotrimer G protein is activated at the same time as the MAPK pathway, inducing the dissociation of the βγ subunit dimer (Ste4 and Ste18) from the G protein α subunit. The βγ subunit triggers the membrane recruitment of a scaffold protein, Ste5, which is able to bind to Ste11, Ste7 and Fus3 [4], [5] [29]. We will neglect the binding of Ste5 to Kss1 which actually exists but has a lower affinity than that of Fus3 [23]. Conversely, during filamentation, to our current knowledge, no scaffold protein is involved. This indicates that Ste5 is a key player in the specificity of the cell response.

The MAPK pathway in *Saccharomyces cerevisiae* is therefore a perfect model to analyze and understand the actual molecular mechanisms of scaffolding.

### Membrane recruitment of scaffold proteins

On the example of the MAPK pathway in *Saccharomyces cerevisiae*, Ste5 is recruited to the membrane when mating is needed. The general importance of membrane recruitment has already been noticed before[30]–[51]. We will show that this simple membrane recruitment of a scaffold protein is sufficient to generate specificity.

Before demonstrating quantitatively this description in the discussion part, it is important to understand qualitatively the underlying principles (see Fig. 1b). Molecules either in the cytosol or bound to the scaffold protein will be randomly activated by a cytosolic activator. First, let's consider the case when a scaffold protein is in the cytosol. Then, molecules whether bound or unbound to the scaffold protein appear to be cytosolic substrates to their activator. Thus, the presence of the scaffold protein does not affect the signal transduction: they are completely invisible for the kinase/substrate molecular couples. Conversely, when recruited to the membrane, a scaffold protein changes the distribution and number of active proteins in the cell.

Such a vision, where the recruitment of the scaffold protein to the membrane is sufficient to induce its biological function, is more efficient and less constrictive than the reactor model. Using the MAPK pathway in yeast, we will show quantitatively the consistency of this picture where the simple membrane recruitment enables scaffold proteins to accurately control specificity, localization and amplification of signaling.

## Discussion

### Distribution of active proteins and specificity

**Figure 3:**
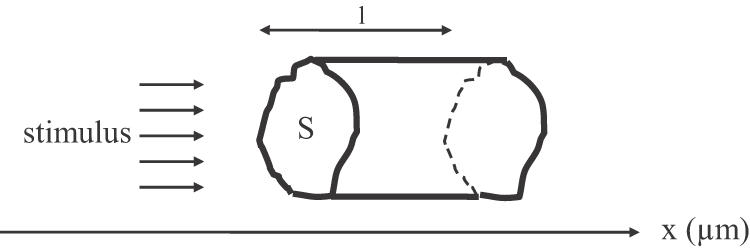
The elongated cell. When a cell receives a signal on a small fraction of its surface, we will consider it as an object that is stimulated on a flat area, S, (where the signal is received). The cell is self similar along the x axis. The volume of the cell is equal to S.l.The shape of S is not important, the only requirement to obtain a cell where the system can be solved in one dimension is that the shape of the cell is self similar by translation along an axis normal to S (the simplest shapes are a cylinder or a parallelepiped shape).

By taking cell models corresponding to simple geometries in which the cell is elongated (cell adhering and stimulated on one side, see Fig. 3) or spherical (cell in suspension), the reaction-diffusion equations can be solved. In these situations, it is possible to work in one-dimension (along the axis for the elongated cell or along the radius for the spherical cell).

Knowing the values of the association and dissociation constants as well as the overall number of each protein, only three parameters remain unknown and can be varied: the three effective affinities of Ste11, Ste7 and Fus3 to Ste5 (see method section). An effective affinity is defined as the ratio between the Ste5-substrate affinity to the fraction of the overall number of membrane recruited Ste5, 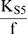. Experimentally, only the Ste5–Fus3 affinity has been measured (1 µM [23]). Thus, the Ste5–Fus3 effective affinity is always above 1 µM. It is also known that Kss1 is more efficiently activated than Fus3 when Ste5 is not membrane recruited [2]. Since Kss1 and Fus3 share the same pathway, the affinity of Kss1 to active Ste7 must be stronger than that of Fus3. In the present calculations, we took Kss1-Ste7 affinity 10 times stronger than Fus3-Ste7 affinity (see method section).

Equation (4) can be analytically solved with the corresponding boundary conditions. Thus, the distribution of active Ste11 was analytically obtained. For the other molecules, numerical solutions of equation (3) were obtained with Mathematica® (Solver NDSolve: it automatically switches between non-stiff Adams method and stiff Gear method).

**Figure 4:**
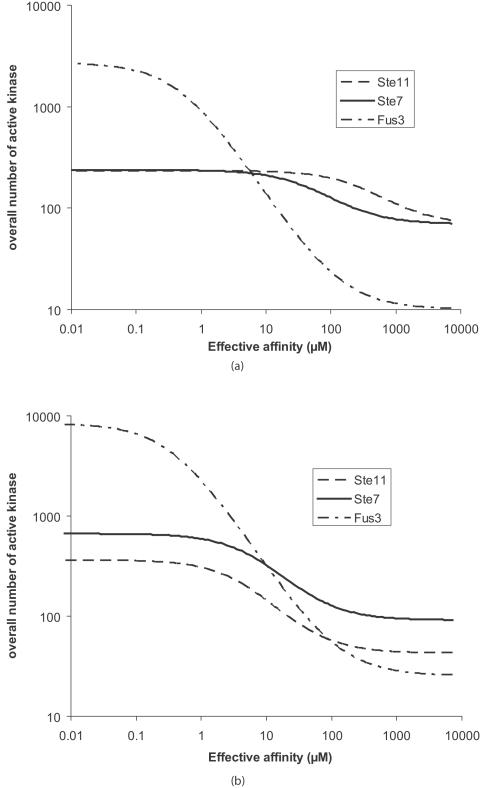
Signal amplification. Overall number of active kinase molecules in the cell as a function of their effective affinity with scaffold protein Ste5. For Ste7, it is supposed that the effective affinity of Ste11 for Ste5 is 1 µM. Similarly, for Fus3, it is supposed that the effective affinities of both Ste11 and Ste7 are 1 µM. (a) Example of an elongated cell that receives a signal on a 2.7 µm^2^ surface area representing about 5% of the overall surface of the cell. (b) Example of a spherical cell that receives a uniform signal over its entire surface.

**Figure 5 pone-0000977-g005:**
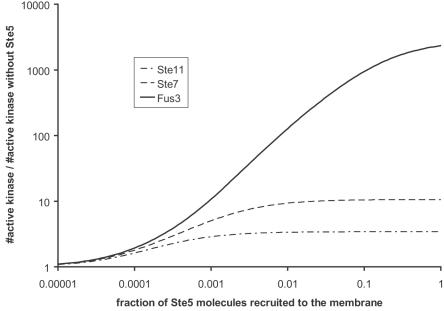
Influence of the number of Ste5 recruited to the membrane. The relative number of active Ste11, Ste7 and Fus3 is plotted with the number of Ste5 recruited at the membrane for a 100 nM affinity between the scaffold proteins and any of the three kinases. When there is no Ste5 these relative numbers are taken equal to 1. The same curves would be obtained for any affinity provided that the effective affinity is kept constant. For instance, the curves for a 1 µM affinity are obtained with an increase by a factor 10 of the fraction of Ste5 recruited to the membrane. Only the case of elongated cells is presented.

The variations of the overall numbers of active proteins with the three effective affinities are given in Fig. 4. For each kinase, it is assumed that the effective affinities of the precedent kinases are 1 µM. It shows a sharp transition close to 10 µM for all three active proteins, Ste11, Ste7 and Fus3: below 10 µM (i.e. stronger bonds and/or more Ste5 recruited to the membrane), the amount of active proteins is significantly increased. This value (10 µM) indicates that the kinase-Ste5 bond does not have to be very strong as long as enough Ste5 is present at the membrane. These curves show that the presence of Ste5 amplifies the signal. In order to better visualize this amplification, it is convenient to plot the increase of active proteins with the fraction of Ste5 recruited to the membrane. Fig. 5 gives the results for affinities equal to 100 nM. It shows that when all Ste5 molecules are bound to the membrane the active kinase populations are multiplied by 3, 10 and 2300 respectively for Ste11, Ste7 and Fus3. For stronger bonds, this effect is even more evident. This shows that the signal is greatly amplified along the pathway by Ste5. At the same time, the number of active Kss1 varies only by a factor 9 as a result of the weak intrinsic affinity of Kss1 and Ste5.

**Figure 6 pone-0000977-g006:**
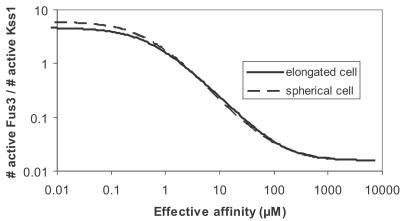
Signal specificity. This curve is a plot of the ratio of the number of active Fus3 over the number of active Kss1 as a function of the effective affinity between the kinases and Ste5 for elongated and a spherical cells. It is assumed that the effective affinities are the same for the three kinases Ste11, Ste7 and Fus3.

**Figure 7:**
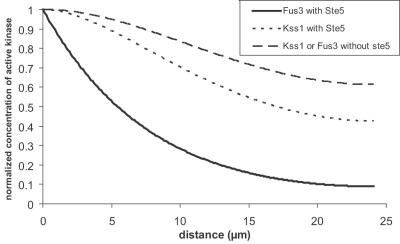
Signal localization. The figure represents the ratio of the concentration of active kinase normalized by the concentration close to the surface that receives the signal as a function of the distance from the membrane. When Ste5 is recruited to the membrane, the polarization of the cell is significantly increased. It is assumed that the effective affinities between the kinases and Ste5 are 1 µM for the three kinases Ste11, Ste7 and Fus3. The localization is not as relevant in the case of a spherical cell since the only polarization that can be achieved is between the vicinity of the membrane and the center of the cell.

The specific response of the cell will be directly correlated to the activation of Kss1 and Fus3. Kss1 will be preferentially active in a non-mating cell while Fus3 will be preferentially active in a mating cell [2]. Thus, in order to check that specificity will be achieved by the membrane recruitment of Ste5, the ratio between active Fus3 and active Kss1 has to be computed. It is displayed in Fig. 6. When Ste5 is not membrane recruited, active Kss1 is dominant (almost 70 molecules per cell, *i.e.* a concentration of the order of 1nM) while the influence of active Fus3 can be neglected (less than 2 active molecules per cell). When Ste5 is recruited to the membrane, Fus3 is much more activated than Kss1 (2600 active Fus3 molecules *vs.* 600 active Kss1 molecules in the plateau of Fig. 6). Furthermore, the presence of Ste5 at the membrane allows a better localization of the targeted kinase (see Fig. 7): active Fus3 tends to be localized close to the membrane where Ste5 is present. More than 80% of the active Fus3 molecules are in the half of the cell close to the area where the signal was triggered. It is known that controlled polarization is extremely important for the response of cells to signals passing by PAKs like the MAPK pathway considered here [52].

The results presented in Fig. 4 to [Fig pone-0000977-g005] [Fig pone-0000977-g006] 7 show that Ste5 enables yeast to specifically use the MAPK pathway during mating in order to massively express active Fus3 molecules in the vicinity of the stimulated area of the membrane. This over activation leads to a significant polarization of the cell and induces the expression of mating genes.

From the distributions of active proteins, it is possible to show that the reactor model cannot be as efficient. In the method section, the average number of Ste5 bound to kinases is estimated. For typical affinities and concentration values, the average number of Ste5 bound to one specific kinase is 19 while the average number of Ste5 simultaneously bound to two kinases is 0.19 (and 0.0019 for three kinases simultaneously bound). These predictions show that there is virtually no Ste5 that are simultaneously bound to two kinases, such as Ste11 and Ste7 or Ste7 and Fus3 since on average there is usually less than one Ste5 molecule that is bound simultaneously to two of the kinases at a given time. It demonstrates that the reactor description is not possible.

### The molecular mechanisms of scaffolding facing the reality of experiments

**Figure 8:**
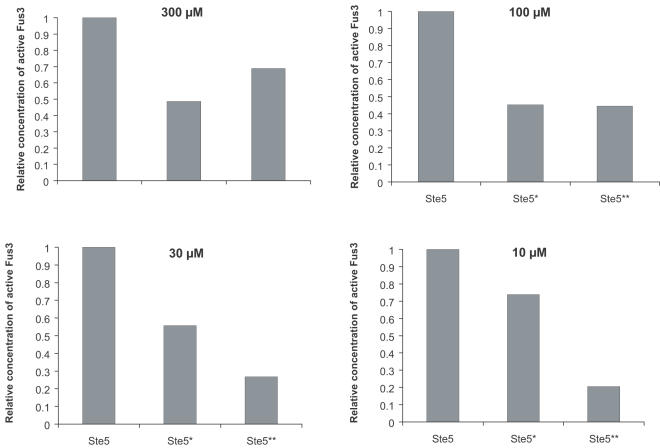
Cell response when mutated Ste5 are recruited to the membrane. Comparison of the total activation of Fus3 with the two mutated Ste5 used in reference [53], [54]. Ste5* (respectively Ste5**) is a scaffold protein where the docking site for Ste11 (respectively Ste7) has been disrupted. For the four effective affinities presented here (10 µM, 30 µM, 100 µM and 300 µM) the concentration of active Fus3 is significantly decreased which could lead to a non functional response to mating pheromones.

Two studies have tried to thoroughly modify the molecular structures in order to evidence the actual means of action of scaffold proteins. These works were performed on the same MAPK as the one mentioned here. In the first one, the authors have removed the binding sites of Ste11 or Ste7 on Ste5 [53]. They observed that the mating response was significantly reduced. This result does not provide an answer in favor of any of the two models presented here. If the reactor model would clearly predict this observation, the simple recruitment of Ste5 to the membrane would give the same result (see Fig. 8). On the other hand, the second set of experiments [54] supports the simple recruitment of Ste5 to the membrane and refutes the reactor model. These authors used mutated Ste5 in which the binding site of a kinase (Ste11 or Ste7) was removed and replaced by another binding group at another location on Ste5. The counterpart of this binding group was attached to the corresponding kinase. In these experiments, Ste5 was still able to bind the Ste11, Ste7 and Fus3, but the locations of the kinase-scaffold protein docking site were different from that in the wild type Ste5. With this system, the mating response still exists. Thus, it is not necessary for the kinases to bind to the scaffold protein in a precise stereochemical orientation. This result is in contradiction with the reactor picture, in which the orientation of the bonds is critical, but is consistent with the simple recruitment of Ste5 to the membrane. These experimental results strongly support the idea that, as predicted here, Ste5 acts by increasing the local concentration of the kinases at the membrane, a job that could just as readily be done by three different membrane-recruited proteins each with one distinct kinase binding site as by one membrane-recruited scaffold with three distinct kinase binding sites.

Another striking feature presented here is that a given Ste5 molecule is unlikely to be bound to more than one of its ligand at a time. The experimental results in reference [54] show that a simultaneous binding of several kinases with the right orientation is not required and therefore tend to validate a much simpler picture where there is no need for more than one kinase to be bound to each Ste5 molecule.

### Universal features and specific evolution

This new vision of the action of scaffold proteins is conceptually very different from the usual one in which they are depicted as reactors where the successive kinases are simultaneously bound in order to meet and activate each other. By creating a source of active kinases at the membrane, a scaffold protein acts more like a triage molecule that directs the pathway toward a specific MAPK at a specific location. Equivalently, it can be considered that the presence of Ste5 at the membrane dramatically increases the local concentration of active kinases.

The description presented here is supported by predictions, by experiments and by the efficiency necessary for the cell survival. As shown in this paper, the resolution of the diffusion-reaction equations predicts this description. Experimentally, the elegant study conducted a few years ago shows that the inhibition of one or two of the binding sites of the scaffold proteins can be compensated for by the recruitment in the same area of another protein with similar sites [54]. These experiments prove that the use of scaffold proteins as reactors is not required for the cell to give the correct response. Therefore, the same signal as the one obtained with Ste5 could be transmitted by recruiting simultaneously three different proteins able to bind respectively Ste11, Ste7 and Fus3. It would be interesting to check experimentally that mating response is preserved when three different membrane-recruited proteins each with one distinct kinase binding site are expressed instead of the scaffold protein. Clearly, the trick of using scaffold proteins makes it much easier for cells: only one protein has to be recruited to the membrane instead of three.

The case of the MAPK cascade in yeast may just be one example among others and it is likely that in the near future, more scaffold proteins using the same basic and simple mechanisms will be found. By their recruitment to the membrane, these proteins allow directing a pathway towards a specific target at a specific location and dramatically amplify the activation of this target. Of course, particular features have been added to each scaffold protein during evolution in order to further increase the specificity. Because life is a constant competition in order to select the best proteins and mechanisms, it is likely that scaffold proteins will always be more efficient than what is predicted in the generic description presented here. For instance, in the MAPK pathway, it has recently been shown that Ste5 is able to induce autophosphorylation of Fus3 [20]. However, specific responses due to scaffold proteins will primarily come from membrane recruitment.

Scaffolding is just one of the basic constituents of the complex signaling code. In the past few years, there have been a lot of efforts in order to conceptualize this complexity and, recently, a global framework has been presented to model cell signaling [55], [56]. The description of other basic constituents that could be integrated in this global framework would be extremely useful for a concrete overall view of signaling codes.

## Methods

### How to simply describe the phosphorylation-dephosphorylation cycle

In the common case of distributive dual phosphorylation of a substrate protein *S* by a kinase *K* [57] there are three intermediate states before reaching the fully phosphorylated active substrate protein *SPP*: (1) the kinase and the substrate are bound, *K-S*, (2) the substrate is phosphorylated a first time, *SP*, and, (3), *SP* is bound to the kinase, *K-SP*. Phosphorylation in the presence of *ATP* is irreversible and it is usually admitted that the newly phosphorylated substrate protein separates immediately from the kinase [29], [58]. This process leads to the following reactions:
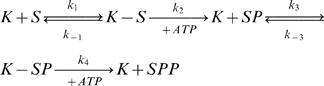
(5)In order to study the phosphorylation/dephosphorylation cycle, it is necessary to also write the dephosphorylation of the phosphorylated substrate *SPP*. For clarity and concision sake, the dephosphorylation process will not be dissected in several intermediate states and will be written:
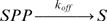
(6)The same approach as the one that is taken for the phosphorylation process would lead to reaction (6).

Serial reaction (5) corresponds to a complicated set of rate equations. The purpose of this section is to detail how a simpler description of the phosphorylation-dephosphorylation cycle can be obtained when a steady state regime is reached and when the concentrations of intermediate species can be neglected. It is often assumed that a steady state regime is reached [7], [24], [26], [27], [51], but it has never been quantitatively checked. Here, we will show how and why the two assumptions, steady state regime and negligible concentrations of intermediate species, are reasonable in many cases.

**Figure 9:**
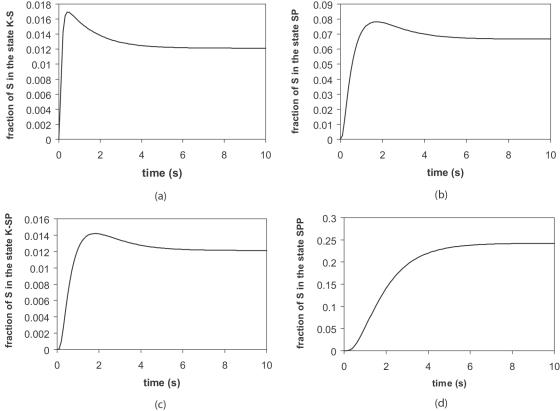
Establishment of the chemical equilibrium. Variation of the concentration of the intermediate states during the phosphorylation process. The curves were obtained by solving equations (7) to (11) assuming a 10 nM concentration of kinase and using the parameters that allow deducing a k_on_ close to the ones used in the rest of text: k_1_ = 0.1 nM^−1^.s^−1^, k_−1_ = k_−3_ = 1s^−1^, k_2_ = k_4_ = 10 s^−1^, k_3_ = 1 nM^−1^.s^−1^. The lifetime of an active molecule should not be less than 1 s for it to be efficient and therefore: k_off_>1 s^−1^. We chose k_off_ = 0.5 s^−1^ in order to obtain a characteristic reaction time larger than reality.

With uniform concentrations over the whole cell, i.e. neglecting diffusion, rate equations can be deduced from serial reaction (5):
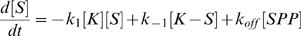
(7)

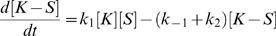
(8)

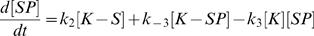
(9)

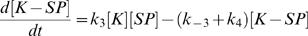
(10)

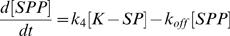
(11)The set of equations (7) to (11) allows the determination of the typical time required to reach equilibrium, the steady-state regime. The variations of the intermediate states concentrations in time (see Fig. 9) show that it takes only a few seconds for the reaction equilibrium to be reached.

Besides, the diffusion of the molecules in the cytosol must be considered. Since the typical distance over which the molecules have to diffuse, L, is of the order of 10 µm and the diffusion constant D of the order of 10 µm^2^s^−1^, the diffusion characteristic time, 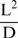, is close to 10 s.

Both reaction and diffusion equilibriums in the cell are reached within a few seconds, which is much shorter than the typical duration of the cell response [28]. We can therefore consider that we are indeed in a steady state regime.

Once the steady-state regime is reached, an estimate of the concentrations of the various intermediate states can be easily obtained from the set of equations (7) to (11) and Fig. 9. This leads to:
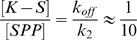
(12)

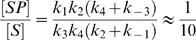
(13)

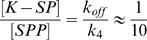
(14)These results show that [SPP] and [S] will be the two dominant species in the solution. It is worth noting that for some values of [K], only one of them may be relevant ([S] for low [K] and [SPP] for high [K]).

[S]+[SPP] can be therefore considered independent of the time and location within the cell in a steady state regime; it is always equal to the initial value of [S], [S]_init_.
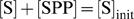
This can be generalized to derive equation (2).

Thus, the assumptions of a steady state regime and negligible intermediate states are realistic. Then, in reaction (5), when the steady state situation is reached, the formation rate of SPP is exactly the same as that of SP because the reaction from K-S to SP is not reversible. Since a fraction 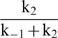 of the K-S molecules are phosphorylated, the formation rate of SPP, k_on_, is equal to this fraction times the K-S formation rate, *i.e.* 
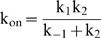
. The reaction can therefore be simplified:
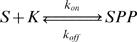
(15)This reaction can be generalized to equation (1) in situations where other intermediate species exist.

### Boundary conditions at the membrane in the MAPK pathway

The set of equations (3) and (4) gives the overall distribution of active proteins owing that the boundary conditions at the membrane are known. These boundary conditions are given by the sources of active proteins at the membrane: for a given active protein, the normal diffusive flow near the membrane is balanced by the formation of the active protein at the membrane.

For simplicity, we will assume that the kinases can be activated in the same manner whether they are bound to Ste5 or not. Then, the presence of cytosolic Ste5 does not affect the reactions and diffusions in the cytosol. On the other hand, when Ste5 is recruited to the membrane, it will change the boundary conditions near this membrane. More precisely, when Ste5 is not recruited (filamentation), only the active Ste11 will be created at the membrane after its phosphorylation by Ste20. The other active proteins do not have any source at the membrane. When Ste5 is present, the story is changed since Ste7 and Fus3 molecules bound to Ste5 can be activated at the membrane. Therefore, the boundary conditions are different when the cell receives a signal for mating and for filamentation. This change in boundary conditions can be seen as a local increase of the concentration of active kinases near the membrane. This is the key point that explains the role of scaffold proteins.

When there is a local surface concentration [Ste5] of scaffold protein, the reaction that takes place at the membrane is:
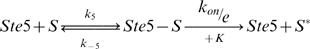
(16)Where S represents any of the substrate kinases to be phosphorylated, and e is the effective thickness over which the Ste5-S complex is accessible. It will usually be of the order of 1 nm (typical size of a molecule).

This reaction leads to the boundary condition:
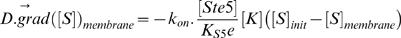
(17)The subscript “membrane” means that the value of the associated parameter is taken at the membrane. K_S5_ is the affinity of S and Ste5: 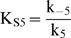. In this paper, we will neglect any association of Kss1 with Ste5, which means that their affinity will be considered extremely large (weak bond).

Equation (17) is only valid for Ste7, Fus3 and Kss1. For Ste11, another contribution has to be added because active Ste20 is only present at the membrane, which leads to the boundary condition:
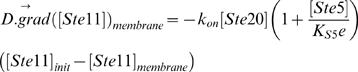
(18)In the membrane area where there is no Ste5, equations (17) and (18) reduce respectively to:
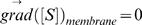
(19)and:
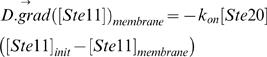
(20)When Ste5 is not recruited to the membrane, equations (19–20) have to be used instead of equations (17–20).

The set of equations (3–4 and 17–20) allows obtaining the distributions of active proteins within the cell.

When k_on_, k_off_, D, e and the initial concentrations are known, the relevant parameter that remains is 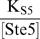 which is proportional to the affinity of Ste5 for the substrate divided by the fraction f of the overall number Ste5 that is recruited to the membrane 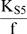. A weak bond with a lot of Ste5 recruited to the membrane can be as efficient as a strong bond with only a few Ste5 recruited to the membrane. 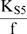 will be called “effective affinity”.

The equations presented in this section can be rewritten for any system involving the recruitment of a receptor molecule to the membrane.

Writing the equilibrium for Ste5-S in reaction (16), it is also possible to get the average number of kinases per Ste5:
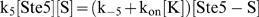
Which means that
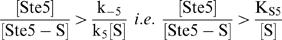
Assuming that all the effective affinities are 1 µM, we obtain (for [S] = 10 nM) that the average number of Ste5 bound to a given kinase is 19 while the average number of Ste5 simultaneously bound to two kinases is 0.19 (and 0.0019 for three kinases simultaneously bound).

### Values of the involved parameters

The values most of the parameters that fully describe the system are known or can be estimated.

First the overall number of each molecule is known [25].

Number of Ste20: 259.

Number of Ste5: 1900.

Number of Ste11: 736.

Number of Ste7: 672.

Number of Fus3: 8480.

Number of Kss1: 5480

The volume of the cell can be well estimated. A spherical *Saccharomyces cerevisiae* cell is assumed to have a 5 µm diameter, corresponding to a 65.45 µm^3^ volume. This is the value taken in this paper.

Therefore, the initial concentrations of the kinases are:

[Ste11]_init_ = 18.7 nM

[Ste7]_init_ = 17.1 nM

[Fus3]_init_ = 215.9 nM

[Kss1]_init_ = 139.5 nM

For the elongated cell (see Fig. 3), the surface over which the signal is received is taken as:

S = 2.7 µm^2^.

The length of the cell is then:

l = 24.2 µm.

The reaction constants in the reaction-diffusion have been estimated before [24], [26], [27]. Because it is known that Fus3 is not much activated without membrane recruitment of Ste5, we decided to choose an association constant of activated Ste7 and Fus3 100 times smaller than that between activated Ste7 and Kss1. The following values were used to solve equations (3) and (4) for the different kinases:

k_on_ = 0.01 nM^−1^s^−1^ for Ste11, Ste7 and Kss1

k_on_ = 0.0001 nM^−1^s^−1^ for Fus3

k_off_ = 0.5 s^−1^

And typical values are given to the diffusion coefficient and the thickness over which a recruited Ste5 acts:

Diffusion coefficient: D = 30 µm^2^.s^−1^

Thickness over which Ste5 is acting: e = 10^−3^ µm.

In the results presented in the main text, it was assumed that all the Ste20 molecules were gathered in the area where the stimulus arrived.
